# Stem Cell Research and Clinical Translation: A Roadmap about Good Clinical Practice and Patient Care

**DOI:** 10.1155/2017/5080259

**Published:** 2017-09-27

**Authors:** Paola Frati, Matteo Scopetti, Alessandro Santurro, Vittorio Gatto, Vittorio Fineschi

**Affiliations:** ^1^Department of Anatomical, Histological, Forensic and Orthopaedic Sciences, Sapienza University of Rome, Viale Regina Elena 336, 00185 Rome, Italy; ^2^IRCCS Neuromed, Via Atinense 18, 86077 Pozzilli, Italy

## Abstract

The latest research achievements in the field of stem cells led in 2016 to the publication of “Guidelines for Stem Cell Research and Clinical Translation” by the International Society for Stem Cell Research (ISSCR). Updating the topics covered in previous publications, the new recommendations offer interesting ethical and scientific insights. Under the common principles of research integrity, protection of patient's welfare, respect for the research subjects, transparency and social justice, the centrality of good clinical practice, and informed consent in research and translational medicine is supported. The guidelines implement the abovementioned publications, requiring rigor in all areas of research, promoting the validity of the scientific activity results and emphasizing the need for an accurate and efficient public communication. This paper aims to analyze the aforementioned guidelines in order to provide a valid interpretive tool for experts. In particular, a research activity focused on the bioethical, scientific, and social implications of the new recommendations is carried out in order to provide food for thought. Finally, as an emerging issue of potential impact of current guidelines, an overview on implications of compensation for egg donation is offered.

## 1. Introduction

Stem cells are the starting point for the development of human organism, representing the precursors of more than 200 cell types. Several studies on stem cells produced increasingly important findings, with considerable implications on the quality of human life, as in the case of the therapies for intractable diseases, the assessment of efficacy and toxicity of new drugs and transplants.

Responding to advances in research and translational medicine, in May 2016, the International Society for Stem Cell Research (ISSCR) published an update of the existing guidelines [1, 2]. Expanding the thematic areas of previous publications, the new guidelines [3] offer interesting insights from an ethical and scientific point of view [4]. The most relevant topics consist of research fairness, based on proven evidence studies and transparency, and patient involvement through effective and truthful communication. The guidelines are inspired by the values already enshrined in key international documents regarding scientific research and experimentation, such as research rigor and integrity, supervision and transparency, patient welfare, and social justice [5–7].

In the first part, the guidelines address the major innovations in the field of research on human embryos and embryonic cells, such as genomic editing, focusing on the usefulness of basic research to expand knowledge on the embryonic development and stating that any attempt to modify the genome of embryos for reproductive purposes is forbidden. Moreover, the area of genome modification is extremely important for the task force, as shown by the rising of the ethical and scientific debate in various countries of the world [8, 9].

For the first time, the ISSCR discusses the issue of human induced pluripotent stem cells (hIPSC). The research on this type of cell is subjected to minor limitations because, despite being very similar to embryonic cells, the IPSC are produced from specialized adult cells (such as skin cells) and cannot therefore be considered as embryonic.

The central points of the discussed guidelines are the transparency and accessibility of data on preclinical and clinical studies as well as the publication of the results in referred scientific journals. According to this view, it is also recommended the need for registration of all clinical trials in progress or carried out in public databases, along with the transparency of the results, in order to ensure a research that is not burdened by fraud attempts. Coherently with several authors, the transparency of the clinical data represents a driver of improved clinical outcomes as well as a valuable tool to overcome the gap between professionals and patients [10, 11].

About the involvement of patients in clinical studies and the increasingly common commitment of the community of patients in funding clinical trials, the guidelines stress the need for these studies to follow the international rules on clinical trials, including supervision by an independent authority which guarantees rigor and scientific validity.

Another interesting aspect of the guidelines is represented by the theme of proper communication and information about the results of scientific research on stem cells. In fact, in setting up this research area as a center of attraction for major funding, it is necessary to ensure the appropriateness of information to prevent the spread of false hopes and unsupported news by accredited and transparent researches [12–15].

The new recommendations invite all the stakeholders involved in the process of communication of scientific data and especially the experts—researchers, clinicians, and industry—to the utmost seriousness in the spreading of data highlighting that scientists must endeavor to promote an accurate, balanced, and effective information and must ensure that the benefits, risks, and uncertainties of stem cell science are not misrepresented. The guidelines support the freedom of clinical research and the right of patients to hope. The ISSCR guidelines, which are valid within the scientific community, are obviously subject to the legislation and regulations of individual nations, although they may provide information on the interpretation of local laws and guidance for research practices that are not covered by the legislation. These principles are to be considered as basics in all countries, and therefore, we must try to standardize the legal systems of all the different states.

## 2. The Updated Guidelines

Although stem cell research and related implications have led over the years to a considerable scientific and popular enthusiasm, the important development of regenerative medicine has determined interesting challenges from an ethical and scientific point of view.

The latest ISSCR guidelines about research in human embryonic cell research and clinical application of stem cells address several issues that are not included in previous versions, with the aim to encompass government regulation, institutional oversight, and communications enclosing basic and translational research (Table 1).

The issues dealt within the latest recommendations are discussed in the following paragraphs in order to highlight the enormous usefulness of the guidelines in the field of research and clinical trials.

## 3. Fundamental Ethical Principles

The variety and importance of ethical considerations regarding research on stem cells is remarkable and, quite possibly, without precedent. The entire path of research is characterized by risks, uncertainties, and problems that require a careful assessment of the foreseeable damages in relation to the expected benefits [16].

In the introductory section of the document, attention is focused on the ethical principles that should guide human stem cell research, clinical translation, and related research activities. In particular, the authors perform a brief discussion about the principles of research integrity, patient welfare, respect for research subject, transparency, and social justice (Table 2).

## 4. Laboratory-Based Human Embryonic Stem Cell Research, Embryo Research, and Related Research Activities

At the core, the new recommendations maintain the notion that the research requires a specialized supervisory process conducted by qualified scientists, ethicists, and community members. This process, defined embryo research oversight (EMRO), aims to review, approve, and monitor all the researches that involve preimplantation stages of human development, human embryos, or embryo-derived cells or entail the production of human gametes in vitro when such gametes are tested by fertilization or used for the creation of embryos. The guidelines exclude the recourse to a specialized EMRO in case of derivation of human pluripotent stem cells from somatic cells via genetic or chemical means of reprogramming (e.g., induced pluripotent stem cells or IPSCs) as long as the research does not generate human embryos or entail sensitive aspects of the research use of human totipotent or pluripotent stem cells.

In the same section of the document, the authors identify three categories of revision to be applied to guarantee that the human embryo and research on embryonic stem cells are proceeding with due consideration, to ensure the coherence of research practices at the international level and specify the nature of the scientific projects that should be subject to review (Table 3).

Furthermore, on the basis of the EMRO process strictness and with the aim of increasing the basic knowledge, the ISSCR expresses its support to the research activities that entail modifying the nuclear genomes of gametes, zygotes, and/or preimplantation human embryos, however prohibiting their application for the purpose of human reproduction due to the limited notions that are currently available.

Due to the growing interest aroused by studies on human-animal chimeras [17, 18], also shown by the recent literature in the field of regenerative medicine [19–21], the authors express a recommendation on the need for a specialized research oversight sustained by scientists and ethicists with relevant expertise on the topic. This advice, besides supporting a rigorous review process for the production of solid scientific evidence, focuses on the ethical implications that are determined by the involvement of animals in scientific research [22–24], with specific reference to the application of the animal welfare principles.

Regarding the procurement of biomaterials, the guidelines update task force sustains a strict review process to be undertaken before the procurement of gametes, embryos, or somatic cells destined to scientific research [25]. A fundamental requirement for the acquisition of biomaterials is the achievement of a valid informed consent. In this context, it is recommended to verify the adequacy of information provided to the research participants [26]. Moreover, an explicit informed consent for the research donation must be obtained separately from the one proposed for the clinical treatment. Since the informed consent document represents a single aspect of the informed consent process, researchers must enrich the dialogue with the providers of biomaterials in the manner that is summarized below (Table 4).

In relation to the activities of derivation of new human embryonic stem cell (hESC) lines, the ISSCR states that these should only be undertaken by qualified scientific personnel and in the presence of a scientific rationale.

A further issue of considerable importance is represented by incidental findings id est the discoveries concerning a research participant or tissue donor that is not directly related to the aims of a study but a carrier of potential implications on an individual's health and reproductive life. In this perspective, the debate on the right to be or not to be informed is still extremely topical [27–31]. The recommendations in this sense urge the development of policies concerning the procedures for communication of incidental findings to research subjects and promote the consultation with the subject on the desire to receive and the ability to select the eventual information.

In agreement with the views that are expressed in the literature, the task force expresses categorically recommending the absolute traceability of the provenance of the stem cell lines and the transparency of data [32, 33].

## 5. Clinical Translation of Stem Cells

The media attention on the field of stem cells together with the numerous successes in animal studies has revealed the need to take stock of the ethical issues related to the clinical translation of knowledge about stem cells [34, 35].

In this regard, the authors have dedicated a whole section to translational medicine focusing on cell processing and manufacture, preclinical studies, clinical research, stem cell-based medical innovation, and clinical application.

About cell processing and manufacture that are subject to local government agencies' regulation, the recommendations highlight the need for an accurate, qualified, and independent oversight review process, to guarantee the best integrity, function, and safety of products. The involvement of donors in the procurement of tissue activity should take place after obtaining a valid informed consent [36] as well as after screening for infectious diseases, risk factors, and, where necessary, genetic diseases. Concerning the cellular derivatives generated from tissue management, the authors promote adherence to the standards of good manufacturing practice (GMP) in accordance with local regulations. The essentiality of an effective manufacture of products finds its foundation in the enormous potential of cell therapies in relation to the satisfaction of the patient's needs, as well as the desirability of promoting the transfer of the product in clinical settings [37].

Another issue that is extensively discussed in the guidelines is represented by preclinical studies, due to their role in the formation of evidence concerning the safety of products and the possible therapeutic efficacy. The evaluation on the safety of cellular products must be made with particular attention to the characterization of the cell lines, the potential toxicity, the possible risk for tumorigenicity, the biodistribution, and the long-term effects of transplanted cells [38]. The effectiveness of interventions based on the use of stem cells must be tested on adequate animal models. In order to guarantee the correct interpretation of the data obtained during the preclinical phase and form meaningful scientific evidence, research actors must ensure the transparency of information making preclinical studies completely accessible whatever the outcomes.

The guidelines also devote a pathway to clinical research promoting the protection of the welfare and rights of people involved in the research, as well as ensuring the integrity of the information resulting from scientific activities. In this regard, it is recommended a constant, independent, and expert oversight who is able to ensure the formation of solid scientific evidence minimizing ethical issues. The conduction of clinical research must be based on the assessment of the evidence supporting the intervention, the analysis of risks and benefits, the acquisition of a valid informed consent, the achievement of a high level of competitiveness in comparison with existing therapies, and the safeguard of human subjects' privacy [39]. The protagonists of clinical trials have to ensure that patients have a conscious and voluntary participation through an approach aimed at improving the effectiveness of information methods [40]. Early phase trials, representing the first opportunity for the assessment of the effects of innovative treatments in humans and being characterized by high levels of uncertainty, imply a particular care in avoiding the overestimation of benefits, ensuring a gradual progression from low-risk to higher-risk experimental conditions and maximizing the scientific value of the tests through a perished and methodical activity. Late-phase trials should provide results of high scientific credibility through the comparison between the new stem cell-based interventions and the best currently available therapeutic approaches; in the absence of treatments of proven efficacy, the comparison should be carried out against placebo or sham comparators. Given the persistence of stem cell treatments, a long-term monitoring of the research subjects' health conditions is appropriate and recommendable. The characterization of the effects of new treatments cannot be separated from the autopsy which, if performed after the acquisition of a valid informed consent, can provide data concerning cellular implantation and functional consequences. Even at this stage, the authors emphasize the importance of the publication of research results regardless of whether they are positive, negative, or inconclusive, as well as the need to report all possible adverse events [41, 42]. About the latter, the implementation must not be limited to the number of reports but rather to the characteristics of adverse reactions such as gravity and predictability [43].

In the section on medical innovations, there is a warning on the marketing of unproven interventions based on stem cell. Specifically, the ISSCR condemns the administration of not demonstrating interventions outside of the context of clinical research or medical innovation compliant with the guidelines and relevant laws, particularly when it is performed as a business activity. The prohibitive approach to the commercialization of unproven products is aimed at the prevention of stem cell tourism, a phenomenon of great social relevance based on the commercialization of treatments that are not supported by scientific evidence [44, 45].

Regarding the clinical application, the appropriateness of new products compared with national regulations and the highest standards of evidence-based medicine assumes a fundamental role [46, 47]. Since the clinical translation does not end with the marketing authorization of the product, at the end of high scientific credibility studies, surveillance on safety and outcomes is recommended, as well as ensuring their accessibility. The centrality of the report activity and patient registries is discussed. Furthermore, on the off-label use of stem cell-based interventions, the authors advise a prudent behavior due to the uncertainties related to this type of treatments.

## 6. Communications

A further issue considered in the discussed guidelines is represented by the communication which, according to the authors, is a crucial element because of the great deal of attention received by the stem cell research from the scientific and political world, as well as the public opinion [48].

On this line, the awareness of the fact that the population coverage and scientific reporting are usually distant from the ideal is emphasized. In fact, coherently with some authors, there are some critical issues regarding the diffusion of overoptimistic information and unrealistic data about the rate of clinical translation by the media [49].

The recommendations are intended to promote a precise, balanced, and understandable communication. In this perspective, according to several authors, the inconsistency of data with the results must be avoided [50–53].

The promotion of adequate communication to the patient both in timing and in methods is essential to improve the quality and safety of care [54]. Information and communication technologies (ICTs) that are used in health care have widely documented the benefits. ICTs include all digital technology platforms capable of supporting the electronic capture, storage, processing, and exchange of data with the objective to promote health.

The virtuous process proposed involves several entities from the scientific community up to political authorities, through professionals, industry, and media communication.

## 7. Standards in Stem Cell Research

In the last section of the document, the ISSCR encourages cooperation between the various research actors aimed at the production of standards for stem cell research and translation, identifying some areas of interest and proposing samples of informed consent documents to promote the standardization process.

The confluence of standards concerning informed consent and procurement of biomaterials, manufacturing regulations, cell potency assays, minimally acceptable changes during cell culture, methods of selection of recipients for novel interventions, reporting of animal experiments, design and reporting of trials, and principles for defining information in datasets generates new opportunities for the advancement of the research and knowledge concerning stem cells.

Furthermore, based on new medical opportunities and ethical challenges in the field of research on stem cells and assisted reproduction techniques, the authors call for a common effort for the periodic review of the recommendations to ensure that science and medical care advances responsibly and ethically.

## 8. Compensation for Egg Donation: ISSCR Point of View

In the section regarding procurement of biomaterials, the Authors made a recommendation on the issue of payments for individuals providing tissue for research. This purpose arises from the need to focus on an ethical debate that has been going on for many years. Ethics, law and policy on payment for oocyte donation has received attention due to the donation of eggs for embryonic stem cell (ESC) research and nuclear transfer. The nature of the payment to women donating oocyte for research has been the subject of international discussion due to the need to find a fair solution for donors.

Despite the possibility of using other sources of supply, the most realistic scenario for getting enough oocytes for embryonic stem cell research and therapeutic needs in the immediate future is constituted by living donors. At the state of the art, the recourse to other techniques such as the use of cadaveric or fetal ovaries requires a much greater amount of knowledge than currently available. Researchers in the field of stem cells have an obligation to carefully supervise this scarce resource and have to make sure that the biomaterials with which they work have been ethically obtained. In this frame, compensation for research subjects represents a commonly accepted concept, regulated by protocols and protective measures, except in cases where the reproductive potential of women is involved [55, 56].

The guidelines very explicitly say that payment should be to compensate, with the final purpose of offsetting nonfinancial burdens for the subject. The concept of compensating women for time, effort, and inconvenience spent on donating oocytes presents considerable attractiveness in terms of justice and psychologic and physical involvement; in particular, compensation for oocyte providers is ethically justified by the philosophical aim of counter-balancing personal losses endured by the research volunteer. The granting of compensation is more justifiable considering the remarkable time, effort, risk of complications, and discomfort sustained by donors. In this regard, it should be remembered how much the egg supply process is difficult, potentially risky, and characterized by a considerable amount of time. Women who undergo these procedures must adhere to a stringent self-administration of drugs for ovarian stimulation, with all the risks associated with hyperstimulation and lack of knowledge about the long-term effects of ovarian stimulation. Throughout the process, which can also be very painful, women must organize their daily lives by ensuring proper drug administration and attend a series of hospital visits to monitor clinical conditions and progress. Furthermore, such compensation is consistent with the consolidated practice of paying participants to biomedical research [57, 58].

On the other hand, as stated by Nuffield Council on Bioethics Report on donation for medicine and research, compensation has to be differentiated by incentive in research ethics, considered as a recruitment tool to encourage subjects to join a study and stay with it until the conclusion so that sufficient data can be collected [59].

An important matter of interest is about the threat of undue inducement and the possibility to enticing socioeconomically disadvantaged women. It is not an ethical insignificant consideration that the offer of compensation does not in itself constitute an undue remuneration, exploitation, or coercion of donors because, even in the absence of payment, such issues tend to occur anyway [60]. As emphasized in the guidelines, undue inducement has to be avoided. According to the latest recommendations, protection of oocyte donors' decisions from the risk of undue inducement should be based on prior ethical review, informed consent, monitoring of recruitment procedures, diligent practice, and careful evaluation of the appropriateness of payment, as in other types of donation. As an adjunct, in view of the nonbinding nature of the guidelines, it would be desirable for local legislators to make acceptable the compensation for egg donation as for other types of research in the biomedical field.

## 9. Conclusions

The potential of interventions based on stem cells generates ethical questions that need to be addressed before research meets its objectives. The latest recommendations of ISSCR appropriately respond to the advancement of knowledge in the field of stem cell confronting emerging issues such as the mitochondrial replacement for assisted reproduction.

Early studies on new biotechnologies are sometimes supported by scientific debatable rationales and inadequate study designs leading to overexcitement for new treatments and unprejudiced commercialization of unproven interventions. In this regard, the statement of ethical principles underlying the scientific activity is an essential guide for researchers, reviewers, and patients.

The most important feature of the latest ISSCR recommendations is to lay the foundation for the protection of the interests of patients, researchers, and industry in order to ensure a productive collaboration in the field of stem cell research and clinical translation.

According to the principles of good clinical practice [61], the guidelines trace precise addresses in order to promote the adoption of measures to maximize the clinical translation and protect research participants.

Ultimately, it seems appropriate to emphasize the importance that the authors give to a central theme such as that of patient information. In this perspective, the recommendations encourage proper information of research participants and patients involved in the various phases of clinical trials. As in other branches of medicine [62], a safe and accurate information is critical to optimize patient participation and prevent the spread of false hopes and news that are not supported by evidence.

In conclusion, despite the achievement of notable successes, the path is still arduous and research requires more and more space as well as a targeted support to the pursuit of increasingly ambitious objectives. Research frequently encounters obstacles of ethical and moral nature that are a counterweight to the impulses that lead science to the threshold of human limitations, as in the case of using stem cells for purposes of eugenics. In this landscape, the analyzed guidelines deal issues regarding stem cell research and clinical translation, providing valid addresses to professionals and their need of periodic update aimed at encouraging the balance between the ethical, scientific, political, and social issues.

What do we learn from these ISSCR guidelines? It is necessary to understand the needs of professionals and support their relationship with patients so to provide complete information about treatments. Try to match technological advances with the needs of patients and facilitate the decision-making process in the selection of innovative therapies and how treatment should be carried out with respect to informed consent [46]. Not at least, besides taking into account the need of constant progression of knowledge in a sensitive area such as that of stem cells, the professionals should also comply with mandatory regulations when available.

## Figures and Tables

**Table 1:** Main issues from ISSCR 2016 “Guidelines for Stem Cell Research and Clinical Translation”.

ISSCR—Guidelines for Stem Cell Research and Clinical Translation
*Fundamental ethical principles*
Integrity of the research enterprise
Primacy of patient welfare
Respect for research subject
Transparency
Social justice
*Laboratory-based human embryonic stem cell research, embryo research, and related activities*
Review processes
Procurement of biomaterials
Derivation, banking, and distribution of human pluripotent stem cell lines
Mechanisms of enforcement
*Clinical translation of stem cells*
Cell processing and manufacture
Preclinical studies
Clinical research
Stem cell-based medical innovation
Clinical application
*Communications*
Public representation of science
Communication about clinical trials
Communication about clinical care
*Standards in stem cell research*
Standard development
Revising ethical guidelines

**Table 2:** Ethical bases of scientific research and translational medicine.

Fundamental ethical principles underlying the new recommendations
*Integrity of the research enterprise*
In order to generate high scientific credibility of evidence, a process of supervision by qualified investigators that ensure the correctness of information is desirable.
*Primacy of patient welfare*
Stem cell research and stem cell-based interventions must be carried out according to the principles of the evidence-based medicine, with the utmost respect for the welfare of research subjects and/or patients.
*Respect for research subjects*
Consent procedures should prevent the overestimation of the potential benefits of the proposed treatment. Subjects with inadequate decision-making capacity must be protected from nontherapeutic procedures that implicate greater than minor increase over minimal risk.
The principle of respect for research subjects should be interpreted more broadly to include other subjects such as tissue providers and researchers who harbor conscientious objections regarding certain aspects of the research.
*Transparency*
Researchers should properly inform the public and the scientific community about the results of trials and preclinical research.
*Social justice*
The successes achieved in the field of clinical translation must be equally and globally distributed with particular regard for disadvantaged populations.

**Table 3:** Review categories recommended to improve the supervision process.

Categories of review proposed for the oversight process
*Category 1. Research that is permissible after review under existing mandates and/or committees and is determined to be exempted from the EMRO process*
This category includes research with established human embryo-derived stem cell lines and research involving the reprogramming of human somatic cells to pluripotency.
*Category 2. Forms of research that are permissible only after review by an EMRO process, possibly in association with other supervisory authorities*
This category embodies activities based on the procurement and use of IVF embryos and human gametes, genetic manipulation of human embryos or gametes, derivation of new pluripotent cell lines from human embryos, creation of human totipotent cells with the potential to sustain embryonic or fetal development, and so forth.
*Category 3. Prohibited research activities*
Research that cannot be pursued due to the lack of a scientific rationale.

**Table 4:** Strategies to implement the informed consent process.

Recommendation to improve the informed consent process
The informed consent dialogue must be carried out in an accurate and transparent manner, especially in the case of involvement of research staff members.
Involvement in the research protocol should be discussed extensively in an interactive and dynamic way.
A counseling service should be available to all donors prior to procurement.
The consent procedures should be updated according to data from research on informed consent as well as, where appropriate, the studies on long-term risks associated with the collection.
